# Magnetic Frustration Driven by Itinerancy in Spinel CoV_2_O_4_

**DOI:** 10.1038/s41598-017-17160-0

**Published:** 2017-12-07

**Authors:** J. H. Lee, J. Ma, S. E. Hahn, H. B. Cao, M. Lee, Tao Hong, H.-J. Lee, M. S. Yeom, S. Okamoto, H. D. Zhou, M. Matsuda, R. S. Fishman

**Affiliations:** 1 School of Energy and Chemical Engineering, Ulsan National Institute of Science and Technology, Ulsan, 44919 Republic of Korea; 2 Department of Physics and Astronomy, Shanghai Jiao Tong University, Shanghai, 200240 China; 3 Key Laboratory of Artificial Structures and Quantum Control, School of Physics and Astronomy, Shanghai Jiao Tong University, Shanghai, 200240 China; 4 Neutron Data Analysis and Visualization Division, Oak Ridge National Laboratory, Oak Ridge, Tennessee 37831 USA; 5 Quantum Condensed Matter Division, Oak Ridge National Laboratory, Oak Ridge, Tennessee 37831 USA; 6 Department of Applied Research and Network R&D, Center for Computational Science and Engineering, Division of National Supercomputing R&D, Korea Institute of Science and Technology Information (KISTI), 245 Daehak-ro, Daejeon, 34141 Republic of Korea; 7 Materials Science and Technology Division, Oak Ridge National Laboratory, Oak Ridge, Tennessee 37831 USA; 8 Department of Physics and Astronomy, University of Tennessee, Knoxville, Tennessee 37996 USA

## Abstract

Localized spins and itinerant electrons rarely coexist in geometrically-frustrated spinel lattices. They exhibit a complex interplay between localized spins and itinerant electrons. In this paper, we study the origin of the unusual spin structure of the spinel CoV_2_O_4_, which stands at the crossover from insulating to itinerant behavior using the first principle calculation and neutron diffraction measurement. In contrast to the expected paramagnetism, localized spins supported by enhanced exchange couplings are frustrated by the effects of delocalized electrons. This frustration produces a non-collinear spin state even without orbital orderings and may be responsible for macroscopic spin-glass behavior. Competing phases can be uncovered by external perturbations such as pressure or magnetic field, which enhances the frustration.

## Introduction

Geometrically frustrated systems have been attracting attention because of their unusual magnetic properties induced by the suppressed conventional long-range magnetic orderings. The spin liquid state has been widely celebrated and theoretically predicted to exist in pyrochlore lattice and spinel systems with nearest-neighbor antiferromagnetic coupling^1^. One more ingredient which makes the playground of the geometrically frustrated systems wider and more interesting is the coupling between the localized spin and itinerant electron in the frustrated systems^2^. The interplay between localized spin and itinerant electron in geometrically frustrated systems is believed to be responsible for many intriguing phenomena such as a metallic spin-liquid state^3^, heavy-fermion behavior^4^, anomalous transport in spin-ice systems^5^, and exotic phases^6–8^. The effect of the interaction between localized spins and itinerant electrons has been investigated intensively on pyrochlores *A*_2_ *B*_2_O_7_ where *A* is rare-earth elements and *B* is transition metals and both *A* and *B* sublattices are frustrated^3–7^. However, it has rarely been explored on another strongly frustrated system, spinel *AB*_2_O_4_, where the magnetic ion *B* is only frustrated.

In the spinels, the degree of the magnetic frustration and the electronic itinerancy on sublattice *B* is a function of the distance of nearest-neighbor *B* atoms (*R*_*B*−*B*_). The shorter distance due to the chemical pressure from a smaller radius of *A* site cation or the physical pressure, the stronger both the electronic itinerancy and magnetic frustration are induced^9^. On the other hand, magnetic *A* site ions can relieve the frustration via the magnetic interactions between the spins on *A* site and the localized spins on sublattice *B*. Therefore, a rich phase diagram due to the strong interplay between localized spins and itinerant electrons can be anticipated when *A* sublattice is a small magnetic ion.

In the spinel vanadates *A*V_2_O_4_ (V^3+^, *S* = 1), the octahedral crystal field on the vanadium site with two electrons in *t*_2*g*_ levels cannot completely remove the degeneracy resulting in the non-quenched orbital angular momentum. The geometrical frustration in many spinel is relieved by lowering the crystal symmetry accompanying non-collinear spin ordering and/or orbital ordering (OO), as an example, from cubic-tetragonal structural transition, which modifies of exchange paths between spins^10–16^. Therefore, OO of partially-filled *d*-electrons on the V site has been observed in many spinel vanadates (*A*V_2_O_4_, *A* = Mn, Fe, Cd, Zn, Mg). Moreover, recent studies suggest that the spinel vanadates become itinerant when *R*_*V*−*V*_ falls around a threshold value of 2.94 Å, which is close to *R*_*V*−*V*_ when *A* = Co (2.97 Å)^17,18^. Since it also has the smallest magnetic *A*-site cation of any known spinel vanadates, CoV_2_O_4_ is the ideal system to study the interplay between itinerancy and localized spins.

The frequency dependence of ac susceptibility and the divergence between the zero-field cooling and field cooling susceptibility at the cubic-tetragonal structural transition indicate a glassy spin state^19–21^. Although the spin-glass behavior of Mn_1−*x*_Co_*x*_V_2_O_4_ is enhanced by Co-doping^9^, CoV_2_O_4_ has higher magnetic ordering temperatures than those of compounds Mn_1−*x*_Co_*x*_V_2_O_4_ with *x* < 1. Indeed, CoV_2_O_4_ has higher para-collinear (*T*_*CL*_) and collinear-non-collinear (*T*_*NC*_) spin transition temperatures than those of any other vanadate spinels. This stands in marked contrast to the pyrochlores^6,7^, where spin-glass phases have lower ordering temperatures with smaller *A* site atom doping. Bizarrely, no structural phase transition with OO has been observed for CoV_2_O_4_^9^ even though the spin structure of CoV_2_O_4_ has been suggested to be non-collinear spin structure from the previous magnetization and neutron diffraction studies^16,19,21,22^. This is sharply contrast to the fact that the most spinels show the structural phase transition concurrent with the orbital ordering and non-collinear spin structure as stated above. Due to these unusual magnetic behaviors, the exotic spin states in CoV_2_O_4_ highly demand a detailed study.

This paper clarifies the origin of the possible non-collinear (NC) spin states of CoV_2_O_4_ without OO and structural phase transition by using density functional theory (DFT) and spin models to interpret neutron-scattering measurements on high-quality CoV_2_O_4_ single crystals. Chemically-driven pressure by Co enhances the itinerancy in CoV_2_O_4_. This itinerancy weakens the OO and strengthens the magnetic and structural isotropies. The frustration fostered by these isotropies^23^ may be responsible for macroscopic glassy behavior in a magnetic field^9,19^. Due to the enhanced frustration, external perturbations such as pressure or magnetic field could uncover novel magnetic phases in cubic CoV_2_O_4_.

## Results

### NC spin states in cubic phase CoV_2_O_4_

**Figure 1:**
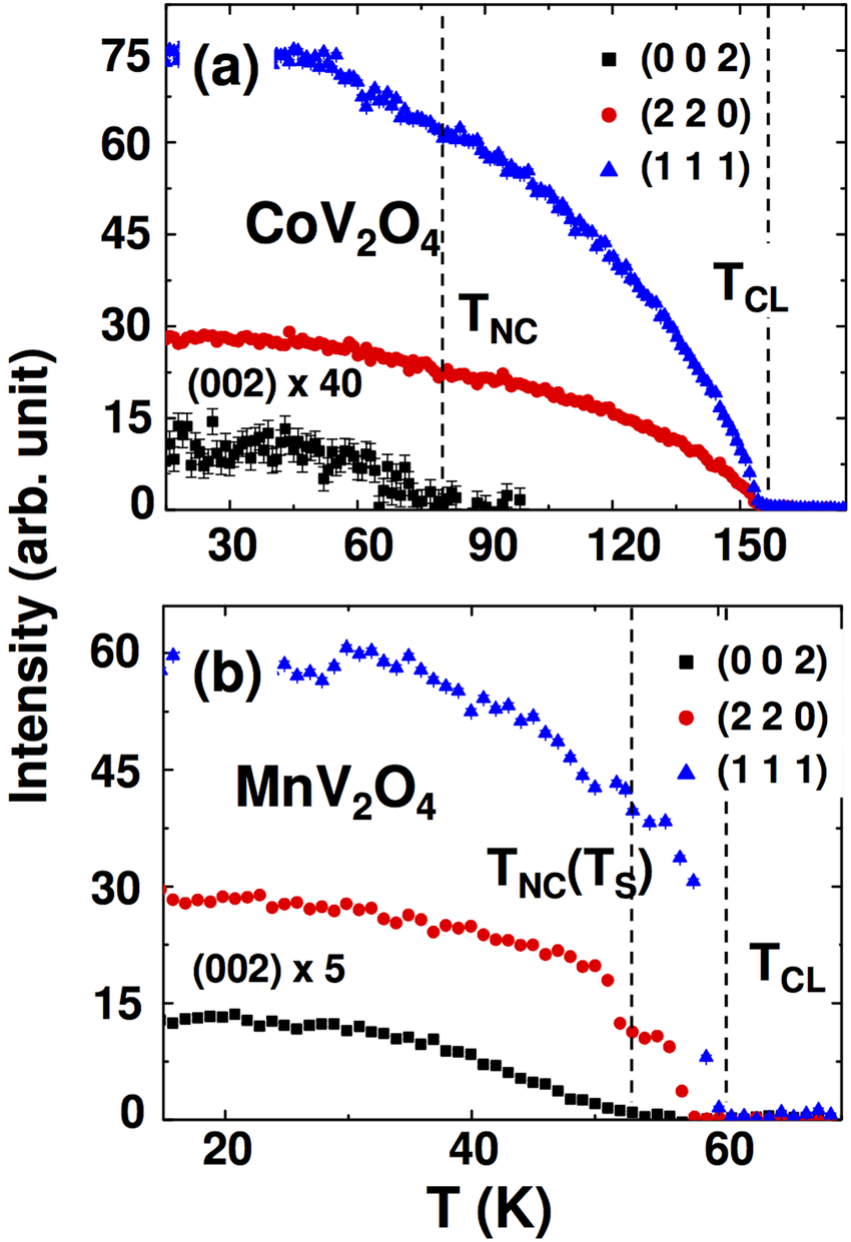
NC spin states in cubic CoV_2_O_4_ compared to tetragonal MnV_2_O_4_. Temperature dependence of the (111) (triangles), (220) (circles), and (002) (squares) Bragg peak intensities for CoV_2_O_4_ (a) and MnV_2_O_4_ (b) measured by neutron diffraction at HB-3A. The peak intensities of (111) and (220) above the magnetic transition temperature are fully from the nuclear structure and were subtracted. The (002) peak is not allowed from the structural symmetry and fully originated from the magnetic scattering. The background was subtracted. All the magnetic peaks observed by our neutron diffraction are instrument resolution limited besides the peak broadening caused by the structural transition for MnV_2_O_4_, and thus indicate the long range ordered magnetic moments. The strongly-reduced intensity of (002) peak in CoV_2_O_4_ indicates that only tiny amount of V spin orders, which is caused by enhanced itinerancy.

To resolve the noncollinear spin structure, we performed the neutron diffraction measurements on cubic CoV_2_O_4_ and tetragonal MnV_2_O_4_ samples. Figure 1 compares the temperature dependence of the (002), (220), and (111) Bragg peaks in cubic CoV_2_O_4_ and tetragonal MnV_2_O_4_. CoV_2_O_4_ and MnV_2_O_4_ show (111) and (220) Bragg peaks rapidly growing below 150 K and 60 K, respectively, denoted as *T*_*CL*_. (111) and (220) Bragg peaks are consistent with the crystal symmetry of these systems, and indicate that collinear ferrimagnetic (FIM) orderings are stabilized. Below 75 K for CoV_2_O_4_, and 57 K for MnV_2_O_4_ denoted as *T*_*NC*_, the intensity of (002) Bragg peaks begin to increase, which is consistent with the previous neutron power diffraction study^21,22^. The (002) peaks are forbidden by structural symmetry. Therefore, the observation of (002) peak indicates the formation of an additional antiferromagnetic (AFM) component in the *ab*-plane, thus a canted spin structure or noncollinear spin structure below *T*_*NC*_^24,25^. In case of MnV_2_O_4_, *T*_*NC*_ accompanies the structural phase transition (*T*_*S*_)^14^. However, no clear structural transition in CoV_2_O_4_ was observed by recent X-ray diffraction and heat capacity measurements^9^. The appearance of Bragg peak intensity at the (002) position is consistent with the recent neutron scattering measurements where it was associated with a two-in/two-out (TI/TO) structure on the V-sublattice^25,26^. Also our DFT calculations later in the paper justify this assumption. Based on the diffraction data, it is valid to assume that the spin configuration of CoV_2_O_4_ is similar to the spin configurations of MnV_2_O_4_^27^, and FeV_2_O_4_^28^.

When the inter-vanadium distance *R*_V−V_ lies around the critical value (2.94 Å)^29^, the system is expected to remain in metallic state down to very low temperatures. Therefore, the effect of the itinerant electrons should be considered in CoV_2_O_4_. Indeed, the ordered magnetic moment refined from the Bragg peaks is 0.47(3)*μ*_*B*_/V in CoV_2_O_4_, which is significantly reduced from 0.95(4)*μ*_*B*_/V in MnV_2_O_4_ due to the increased itinerancy of electrons. In addition, *T*_*NC*_ of CoV_2_O_4_ is higher than that of MnV_2_O_4_ because of a new exchange interaction originating from the itinerant electrons, which we will clarify later with the calculated band structures. An additional magnetic frustration among the localized spins may be indirectly induced by electronic itinerancy as well. Curiously, the structural phase transition associated with OO commonly observed in many vanadates is absent in CoV_2_O_4_, even though the TI/TO seems to emerge robustly. Since the TI/TO state originates from OO in tetragonal compounds^27,28^, the isosymmetric TI/TO state in cubic CoV_2_O_4_ without any OO must have a different origin associated with its itinerancy and frustration.

### Single-ion anisotropy suppressed by itinerancy

**Figure 2:**
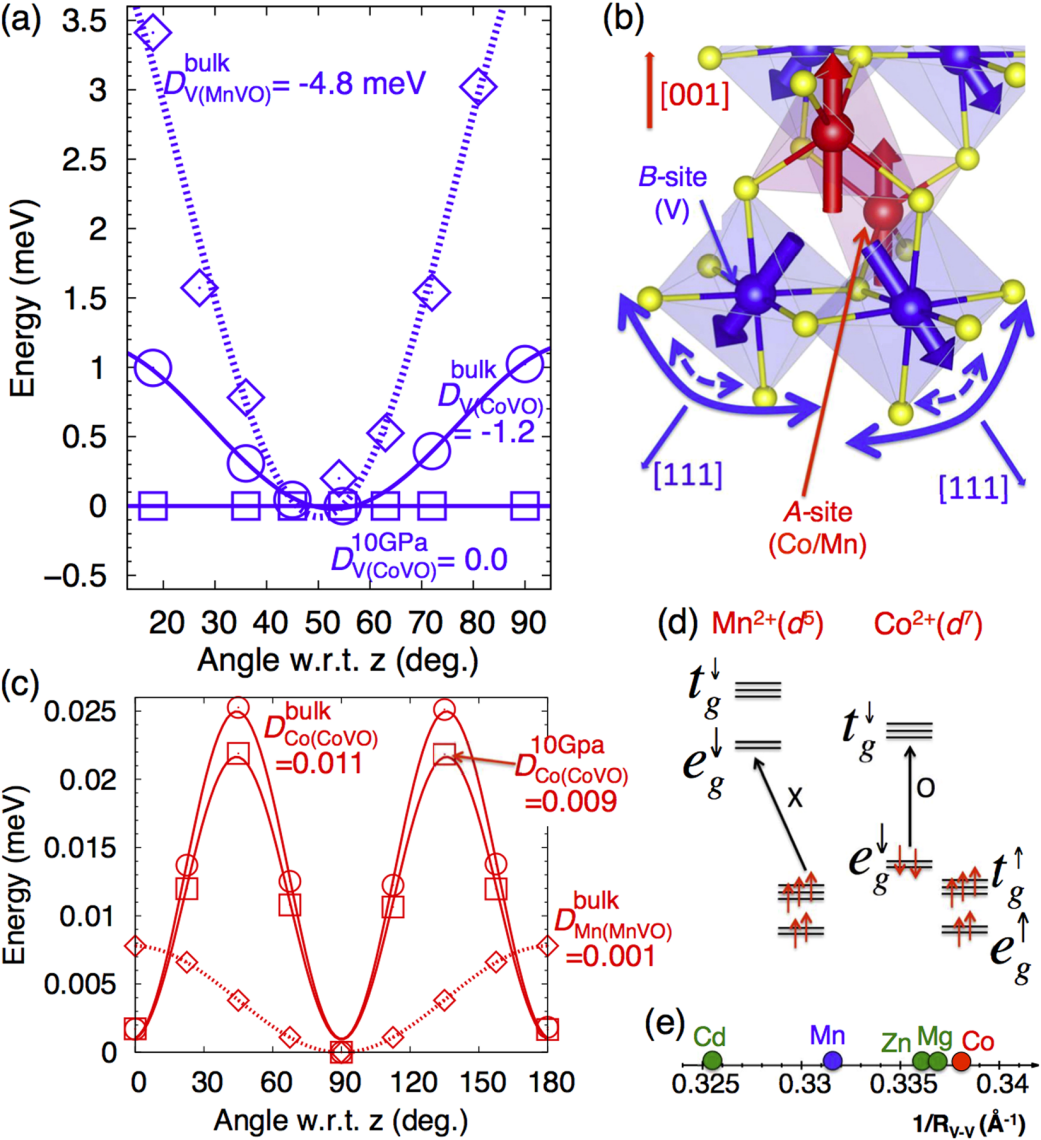
Reduced single-ion anisotropy (SIA) of V in CoV_2_O_4_ compared with that in MnV_2_O_4_. (a) Total energy versus angle and associated SIA (meV) of V^3+^ in ambient (circle) and 10 GPa (square) pressure for bulk CoV_2_O_4_ and MnV_2_O_4_ (diamond). (b) NC spin configurations of V^3+^ and Co^2+^/Mn^2+^ spins pointing along local [111] and global [001] directions, respectively. The round bold (dotted) arrows close to V spins depict the rotational flexibility in CoV_2_O_4_ (MnV_2_O_4_). (c) SIA of Co^2+^ in bulk CoV_2_O_4_ under 10 Gpa compared to SIA of Mn^2+^ in bulk MnV_2_O_4_. (d) Orbital occupation configuration of Mn^2+^ (*d* ^5^) and Co_2+_ (*d* ^7^). (e) *R*_*V*−*V*_ in CoV_2_O_4_ and MnV_2_O_4_ are compared with *R*_*V*−*V*_ in other vanadates from ref.^29^.

First-principles calculations were used to explore the microscopic origin for the complex NC state in cubic CoV_2_O_4_. As shown in Fig. 2(a) and (c), the major magnetic anisotropy appears on the V^3+^ site with a magnitude two or three orders larger than that for the *A*-site (Co/Mn). Although the V^3+^ ions are surrounded by similar octahedra in both CoV_2_O_4_ and MnV_2_O_4_, the local [111] single-ion anisotropy (SIA) of V^3+^ is significantly reduced in CoV_2_O_4_ (−1.2 meV) compared to that in MnV_2_O_4_ (−4.8 meV) due to the melting of OO by the pressure-induced itinerancy in CoV_2_O_4_. Calculated by DFT, the SIA on the V sites totally disappears with an external pressure around 10 GPa in CoV_2_O_4_.

While the AFM V-V interaction in a pyrochlore lattice with local [111] SIA favors the all-in/all-out (AI/AO) spin structure, the disappearance of SIA fosters strong magnetic frustration^23^. OO relieves the frustration in MnV_2_O_4_ with structural phase transitions. However, the frustration reappears in CoV_2_O_4_ due to the melting of the OO and the suppression of the easy-axis anisotropy by itinerancy, as shown in Fig. 2(a,b). The recovered frustration might be responsible for the macroscopic spin-glass behavior^9^ below *T*_*NC*_ due to the competing ground states^30^.

**Figure 3:**
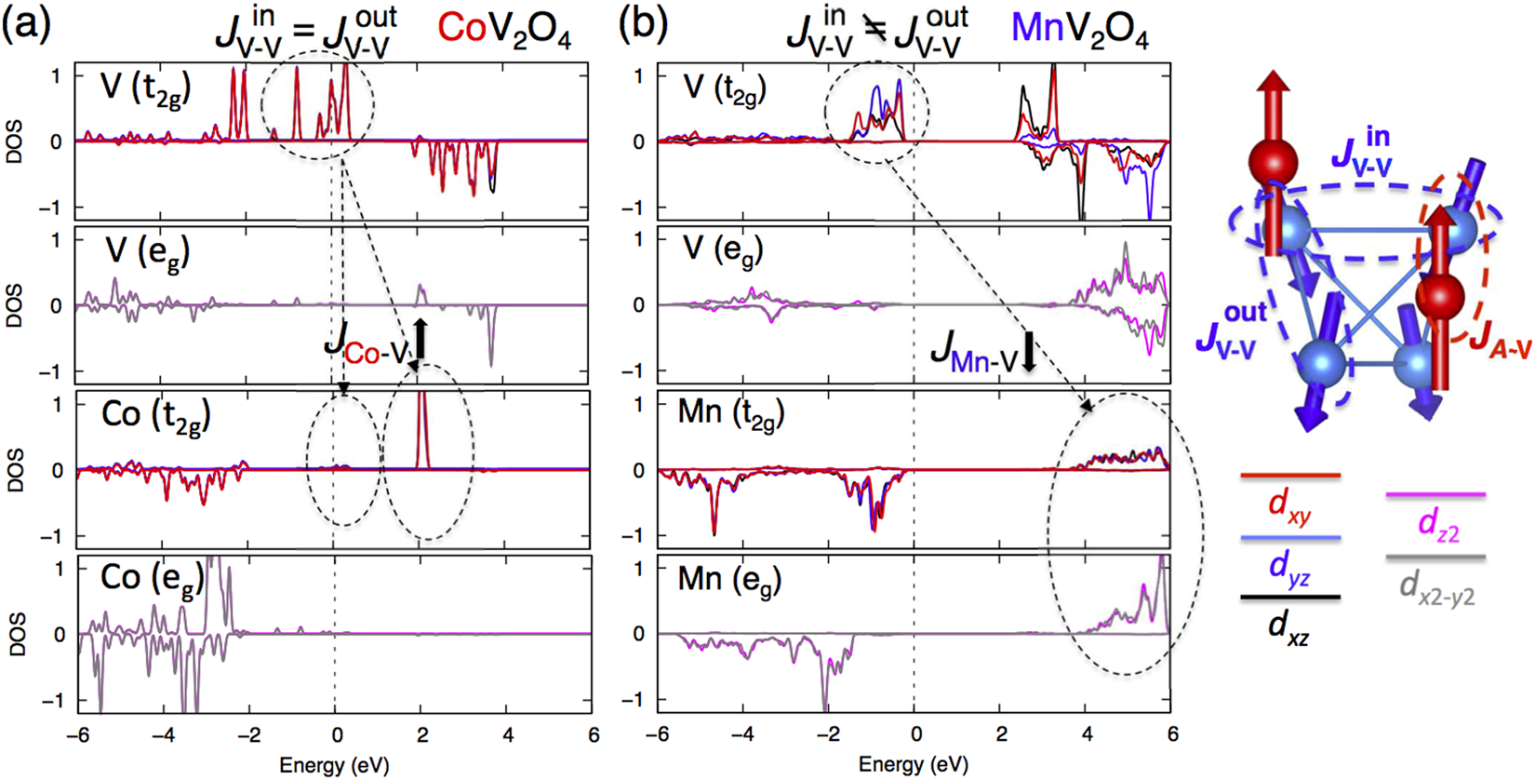
Origin of the enhanced magnetic ordering temperature in CoV_2_O_4_. Projected density-of-states of CoV_2_O_4_ **(a)** compared to MnV_2_O_4_ **(b)** in unit of eV^−1^. Dotted arrows denote the energy differences, Δ between V and Co/Mn for possible AFM super-exchange (*J*_A−V_ ~ −*t* ^2^/Δ). *t* is the hopping parameter between orbitals.

The SIA of *A*-site (Fig. 2c), (A = Co, Mn) is quite negligible compared to the SIA of V^3+^. While Mn^2+^ has a weak easy-plane axis because of the compressed tetragonal structure (*c*/*a* < 1), Co^2+^ does not exhibit anisotropy because of the isotropic cubic structure. The SIA of Co^2+^ is much less dependent on pressure than that of V^3+^ since Co^2+^ electronic states lie significantly below the Fermi energy (*ε*_*F*_) and are thereby electronically encapsulated, as shown in Fig. 3(a). Only V^3+^ states cross *ε*_*F*_. Therefore the pressure-induced itinerancy will only affect the spins on V^3+^ sites. The moment of Co ion is not affected by itinerancy.

### Enhanced exchange couplings

**Figure 4:**
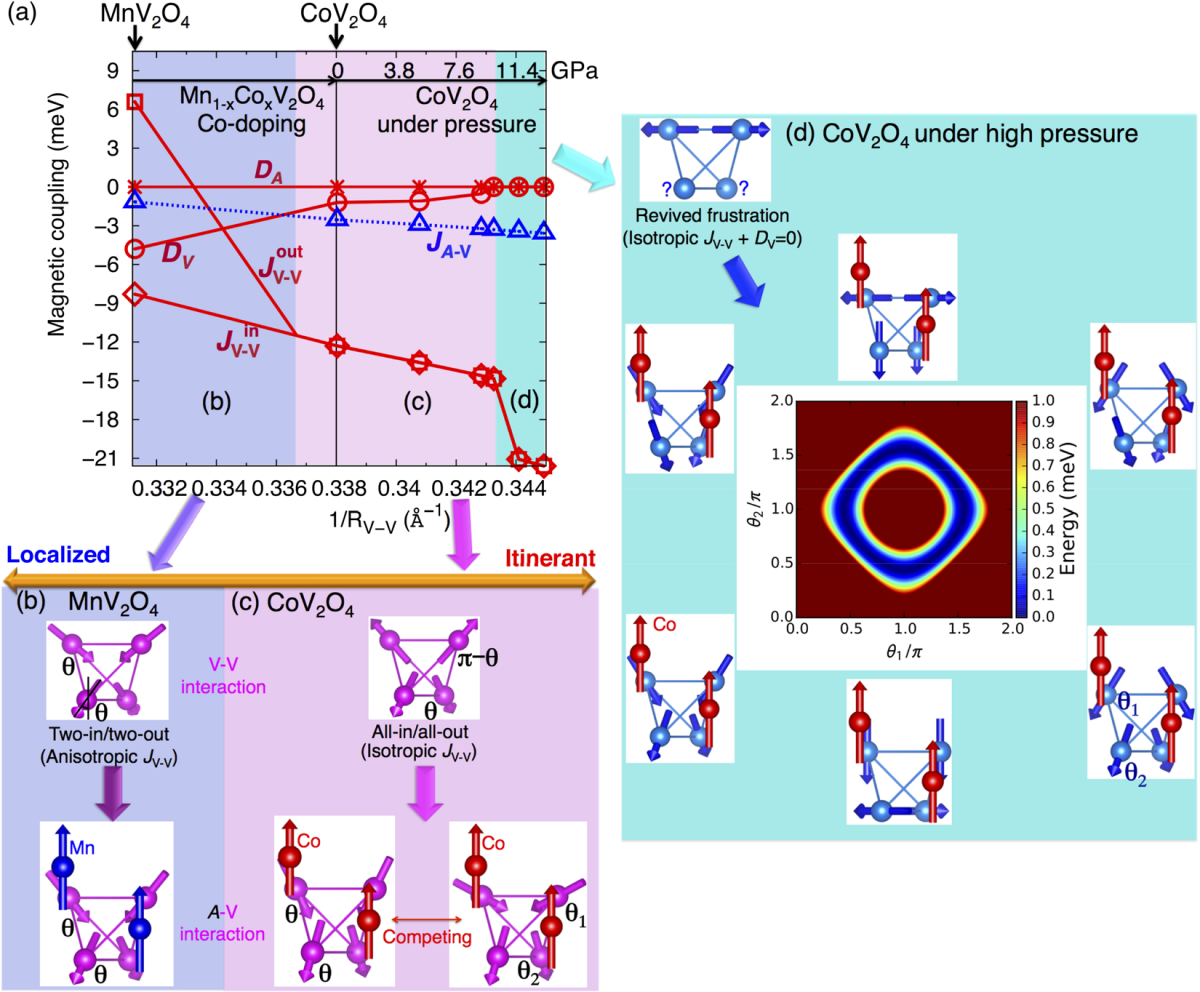
Evolution of magnetic couplings and competing ground states driven by Co-doping and pressure. (**a**) Change of all magnetic interactions with Co doping and external pressure (GPa) calculated by LSDA + *U* for the ground states at zero temperature. Points represent DFT results and the connecting lines are a guide for eye. ${J}_{{\rm{V}}-{\rm{V}}}^{{\rm{in}}}$ and ${J}_{{\rm{V}}-{\rm{V}}}^{{\rm{out}}}$ are expected to be degenerate at *x* = 0.8 (cubic) in Mn_1−*x*_Co_*x*_V_2_O_4_ from experiments^9,24^. Bold (dotted) lines represent the exchange (*J*) and SIA (*D*) interactions. (**b**) The anisotropic *J*_*V*−*V*_ prefers the TI/TO state and the antiferromagnetic *J*_*Mn*−*V*_ also stabilizes TI/TO state in Mn-rich region. (**c**) The isotropic *J*_*V*−*V*_ prefers the AI/AO state but the antiferromagnetic *J*_Co−V_ tries to stabilizes the TI/TO state in Co-rich region. Thus, two states of the isosymmetric one-angle TI/TO state and the two-angle state that evolves from the AI/AO state compete with each other. **(d)** The energy landscape of CoV_2_O_4_ with the disappearance of SIA at high pressures. The massive degeneracies in the energy landscape of two angles (*θ*_1_ and *θ*_2_) may induce spin glass or liquid phases as explained in the text.

As shown in Fig. 2 in Supplementary material, the Bragg peaks do not split or broaden with decreasing temperature below 100 K, indicating the absence of a structural transition. In agreement with this measurement, DFT calculations confirm the structural isotropy (*c*/*a* = 1) of CoV_2_O_4_. As shown in Fig. 3, the *t*_2*g*_ (*d*_*xy*_ = *d*_*yz*_ = *d*_*xz*_) and *e*_*g*_ (${d}_{{z}^{2}}$ = ${d}_{{x}^{2}-{y}^{2}}$) electronic levels become equally occupied and degenerate in cubic CoV_2_O_4_. The structural and electronic isotropies also produce the same exchange interactions ${J}_{{\rm{V}}-{\rm{V}}}^{{\rm{in}}}$ = ${J}_{{\rm{V}}-{\rm{V}}}^{{\rm{out}}}$ = −12 meV between all spins on the tetrahedron as calculated from first principles, Fig. 4, where ${J}_{{\rm{V}}-{\rm{V}}}^{{\rm{in}}}$ = ${J}_{{\rm{V}}-{\rm{V}}}^{{\rm{out}}}$ are in-plane and out-of-plane exchange interaction, which are not equivalent in tetragonal phase (the right panel of Fig. 3). These coupled structural, electronic, and magnetic isotropies foster frustration and the observed NC phase in Fig. 1.

Comparing the densities-of-states of CoV_2_O_4_ and MnV_2_O_4_ reveals the origin of the enhanced magnetic ordering temperatures in CoV_2_O_4_.The large energy difference (~5 eV) between the occupied V and Mn *d* states weakens the exchange between Mn and V. By filling the *e*_*g*_ minority spin levels as indicated in Figs 2(d) and 3(a), Co significantly lowers the *t*_2*g*_ unoccupied energy level and enhances the exchange interaction between Co and V. DFT calculations reveal that the magnitude of the AFM *J*_A−V_ is twice as large in CoV_2_O_4_ (−2.5 meV) as in MnV_2_O_4_ (−1.2 meV). As reflected by the neutron-scattering measurements in Fig. 1, the enhanced *J*_A−V_ causes *T*_*CL*_ to more than double in CoV_2_O_4_ (150 K) compared to MnV_2_O_4_ (53 K).

Surprisingly, the induced itinerancy also increases the NC ordering temperature even without OO in CoV_2_O_4_. As shown in Fig. 1, *T*_*NC*_ significantly increases in CoV_2_O_4_ (75 K) compared to MnV_2_O_4_ (57 K). Although it exhibits the higher NC ordering temperature, CoV_2_O_4_ also exhibits glassy behavior^9,19^. While the reduced SIA and induced isotropies foster frustration^23^, the enhanced exchange interaction relieves the frustration and enhances the ordering temperatures. In the series of Mn_1−*x*_Co_*x*_V_2_O_4_, the spin-wave gap (~2 meV) remains relatively unchanged with Co-doping (*x*)^24^ despite the enhanced magnetic ordering temperatures proportional to *J*_A−V_. Since the spin-wave gap is proportional to $\sqrt{{D}_{{\rm{V}}}\times {J}_{{\rm{A}}-{\rm{V}}}}$, the increase in $|{J}_{{\rm{A}}-{\rm{V}}}|$ is compensated by the reduction in the anisotropy *D*_v_ in CoV_2_O_4_. By enhancing both competing effects (itinerancy-driven isotropies with reduced SIA and strengthened exchange), Co doping can foster various novel states in CoV_2_O_4_.

### Novel phases induced by frustration

The comparison between CoV_2_O_4_ and MnV_2_O_4_ in Fig. 4 reveals the origin of the NC states in CoV_2_O_4_. The key handle to tune the magnetic couplings is the distance between the V atoms (*R*_V−V_ along the *x*-axis) controlled by chemical doping and external pressure. In MnV_2_O_4_, the OO of the V ions relieves the magnetic frustration of the pyrochlore lattice and stabilizes the TI/TO NC spin state. The AFM Mn-V interactions increase the canting angle while maintaining this TI/TO state (Fig. 4(b)). By introducing itinerancy, Co doping promotes isotropic V-V interactions and favors the AI/AO spin state. Within the tetrahedron network, the AI/AO state has two distinct canting angles *θ* and *π*-*θ* compared to the one canting angle *θ* of the TI/TO state, where the canting angle *θ* is a measure of deviation of spin from the −*z* axis as shown in Fig. 4(b). Guided by the DFT parameters for CoV_2_O_4_, our model calculation indicates that the new two-angle state based on the AI/AO state lies within 0.1 meV/unit-cell of the TI/TO ground state. The isotropic exchange (${J}_{{\rm{V}}-{\rm{V}}}^{{\rm{in}}}={J}_{V-V}^{{\rm{out}}}$) fosters a new two-angle AI/AO structure that can be stabilized by a magnetic field.

External pressure may also increase the degree of frustration. For high external pressure^9,17^ ~10 GPa, the enhanced itinerancy fully suppresses the local SIA (*D*_v_ ~ 0) of V as in Fig. 4(a) and revives the magnetic frustration of the pyrochlore lattice. Although AFM exchange between the Co and V sites then induces the observed isosymmetric TI/TO spin structure, the frustration fostered by itinerancy and the alternative states that compete with the TI/TO ground state are possibly responsible for the measured magnetic anomalies^17^ and spin-glass behavior^9^. Moreover, the absence of SIA (*D*_v_ ~ 0 and *D*_A_ ~ 0) at high pressure above 10 GPa (Figs 2(a) and 4(a)) preserves the rotational symmetry and may stabilize a continuum of degenerate states where the two angles (*θ*_1_, *θ*_2_) rotate without energy cost as obtained in the Appendix and shown in the energy landscape of Fig. 4(d). This massive degeneracy can induce spin-glass or spin-liquid-like behavior. Since neutron scattering is limited to relatively low pressures, these novel states should be studied with synchrotron magnetic X-ray scattering.

### Magnetic field measurement

**Figure 5:**
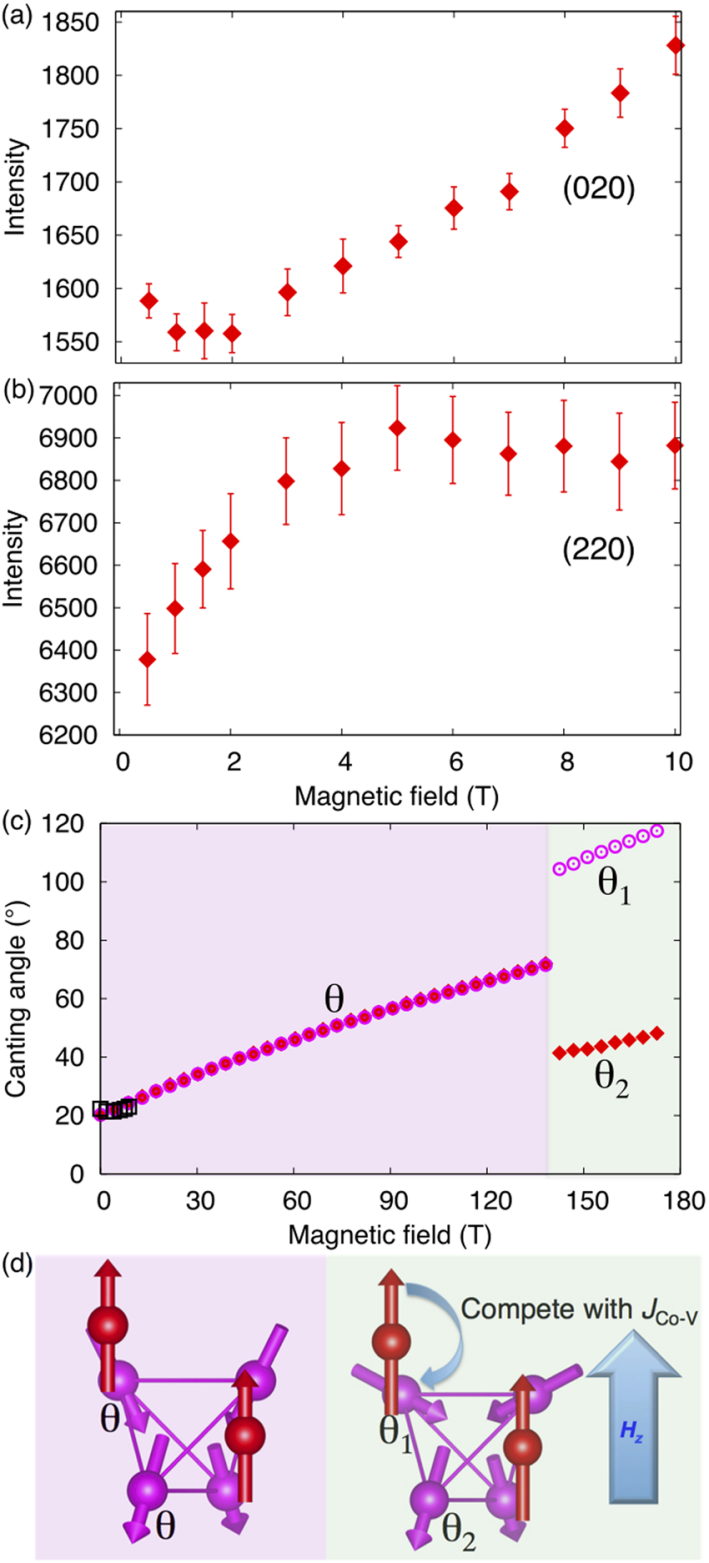
Prediction of novel phases driven by magnetic field in Mn_0.2_Co_0.8_V_2_O_4_. Experimental results for the field-dependent (020) (**a**) and (220) (**b**) Bragg peak intensities. (**c**) Field dependence of spin angle *θ* calculated by spin models using DFT parameters. First-order phase transition from TI/TO to AI/AO-derived state with magnetic field is indicated by the jump in *θ*. Black open squares are from the neutron scattering measurement up to 10 T. (**d**) One-angle state based on TI/TO (left) and two-angle state based on AI/AO (right). The latter is driven by the competition between *J*_Co−V_ and magnetic energy.

Figure 4(a) provides a guide to uncover the novel states produced by the regenerated frustration. Although all other magnetic couplings (isotropic *J*_*V*−*V*_, reduced *D*_v_) foster frustration (bold red line), the remnant AFM interaction *J*_Co−V_ (dotted blue line in Fig. 4(a)) still relieves frustration. An external magnetic field ($\overrightarrow{H}=H\hat{z}$) can help restore frustration by weakening *J*_Co−V_, thereby inducing the novel two-angle state of CoV_2_O_4_, as shown in Fig. 5.

To check the effect of a magnetic field on CoV_2_O_4_, we carried out further elastic neutron-scattering measurements on the Co-rich single-crystal spinel Co_0.8_Mn_0.2_V_2_O_4_, which preserves the cubic structural and magnetic isotropies as in Fig. 4(a) but exhibits stronger scattering intensity than CoV_2_O_4_ due to the larger size of the single crystal. The V^3+^ AFM components in the *ab*-plane increase with the magnetic field (*H*_*z*_ > 3 T), as indicated by the increased intensity of (020) (see Fig. 5(a)). At *H*_*z*_ < 3 T, the increased intensity of (220) reflects the reorientation of the magnetic domains; at *H*_*z*_ > 3 T the (220) intensity is saturated, indicating that all magnetic domains are fully oriented and that the FIM components are constant. This is consistent with magnetization measurements on a polycrystalline sample, which provide a saturation field of ~2 T^19^. Note that the magnetic components for ${M}_{z}^{{\rm{Co}}}$ and ${M}_{z}^{{\rm{V}}}$ both contribute the magnetic scatterings at (220). Considering the Co magnetic moments saturate ferromagnetically at zero field, we assume ${M}_{z}^{{\rm{Co}}}$ does not change with the field. Therefore, no change of the (220) intensity at fields above 3 T indicates ${M}_{z}^{{\rm{V}}}$ stays almost constant at the field between 3 T and 10 T. The small change in ${\rm{\Delta }}{M}_{z}^{{\rm{V}}}={M}^{{\rm{V}}}\mathrm{(0.6}{\mu }_{B})\cos \,{21}^{\circ }({\rm{at3T}})-{M}^{{\rm{V}}}\mathrm{(0.6}{\mu }_{B})\cos \,{24}^{\circ }({\rm{at10T}}) \sim 0.01{\mu }_{B}$, which is negligible compared with $\Delta {M}_{xy}^{{\rm{V}}}$ and the resolution limit of ~0.1 *μB*. So we can safely assume that both ${M}_{z}^{{\rm{V}}}$ and ${M}_{z}^{{\rm{C}}o}$ are constant above 3 T. Since the AFM components of V^3+^ in the *ab*-plane (${M}_{ab}^{{\rm{V}}}$) continue to grow above 3 T, the canting angle ($\theta ={\tan }^{-1}[{M}_{ab}^{{\rm{V}}}/{M}_{z}^{{\rm{V}}}]$) of the V^3+^ spins must increase with the magnetic field along [001].

Using the spin model (Eq. 1) combined with DFT parameters (Fig. 4(a)), we confirm the increase in the canting angle with magnetic field in Fig. 5(c). The two-angle AI/AO state has an energy within 0.1 meV/unit-cell of the the one-angle TI/TO ground state in the Co-rich region. We predict that this new state is stabilized by a large magnetic field of about 140 T, as shown in Fig. 5(c),(d). Although only the one-angle TI/TO state was previously reported in vanadate compounds (*A*V_2_O_4_, *A* = Zn, Mn, Fe), various competing states appear in CoV_2_O_4_ due to frustration. It is likely that those states can be revealed by a magnetic field or pressure.

Of course, the critical magnetic field (*H*_*Z*_ = 140 T) is too large for neutron scattering measurements. However, the first-order phase transition from the one-angle to the two-angle state may be captured by magnetic susceptibility measurements. Moreover, various methods can be employed to reduce the critical field. Since external pressure suppresses SIA and revives frustration as discussed in the previous section, pressure may also reduce the critical magnetic field. Contrary to the usual expectation, a magnetic field may strengthen frustration and noncollinearity in CoV_2_O_4_ by competing with the only exchange coupling (*J*_Co−V_) that hampers frustration.

## Discussion

It is natural to wonder if cations significantly smaller than Co^2+^ such as Be^2+^ can be substituted on the *A*-site to induce even more itinerancy and consequent frustration. However, a non-magnetic *A*-site reduces the magnetization of V so much that the system would become paramagnetic^29^. Because strong magnetic interactions between the *A* and *B* sites is required, Co is the only candidate *A*-site cation to support localized spins with enhanced *J*_Co−V_ while also promoting itinerancy on the *B* site.

Compared to other vanadates (*A*V_2_O_4_), the frustration in magnetically and structurally isotropic CoV_2_O_4_ explains its NC and macroscopic spin-glass properties. Since the AFM interaction between Co and V is the only factor that relieves the magnetic frustration, weakening the AFM interaction by a magnetic field or further reducing the SIA by external pressure can rekindle the frustration and reveal alternative states. Among spinel vanadates, CoV_2_O_4_ is uniquely located at the crossover between localized and itinerant behavior. Consequently, many exotic properties and new phases can be produced by restoring the frustration of the pyrochlore lattice.

## Method

### Sample preparation

Single crystals of CoV_2_O_4_, Co_0.8_Mn_0.2_V_2_O_4_ and MnV_2_O_4_ were grown by the traveling-solvent floating-zone (TSFZ) technique. The feed and seed rods for the crystal growth were prepared by solid state reaction. Appropriate mixtures of MnO, CoCO_3_, and V_2_O_3_ were ground together and pressed into 6-mm-diameter 60-mm rods under 400 atm hydrostatic pressure, and then calcined in Ar at 1050 °C for 15 hours. The crystal growth was carried out in argon in an IR-heated image furnace (NEC) equipped with two halogen lamps and double ellipsoidal mirrors with feed and seed rods rotating in opposite directs at 25 rpm during crystal growth at a rate of 20 mm/h.

### Neutron-scattering experiments

Single-crystal neutron diffraction was performed to determine the crystal and magnetic structures using the four-circle diffractometer (HB-3A) at the High Flux Isotope Reactor (HFIR) of the Oak Ridge National Laboratory (ORNL). A neutron wavelength of 1.003 Å was used from a bent perfect Si-331 monochromator^31^. High magnetic field single-crystal neutron diffraction experiments were performed on the cold neutron triple-axis spectrometer (CTAX) at HFIR, ORNL. The incident neutron energy was selected as 5.0 meV by a PG (002) monochromator, and the final neutron energy was also set as 5.0 meV by a PG (002) analyzer. The horizontal collimation was guide-open-80′-open. Contamination from higher-order beams was removed using a cooled Be filter. The scattering plane was set in the (H,K,0) plane and the magnetic field was applied perpendicular to the scattering plane. The nuclear and magnetic structures were refined with the program FULLPROF^32^. We examined more than 18 peaks of lattice and magnetic Bragg peaks to extract the precise nuclear and magnetic structural information as shown in the S2 in the Supplementary material. Due to the domain re-orientation effect, Bragg peak intensities of both (220) and (020) diffractions increase sharply in small magnetic fields, but the (220) diffraction is saturated above about 3 T. The Bragg peak intensity of the (020) diffraction, corresponding to the magnetic component of V in the *ab*-plane, inceases linearly with field.

### First-principles calculations

First-principles calculations were performed using density-functional theory within the local spin-density approximation with a correction due to on-site Hubbard interaction (LSDA + *U*) as implemented in the Vienna *ab initio* simulation package (VASP-5.3)^33^. We used the Liechtenstein^34^ implementation with on-site Coulomb interaction *U* = 6.0 eV and on-site exchange interaction *J*_*H*_ = 1.0 eV to treat the localized 3d electron states in Co, Mn, and V; this choice of *U* is close to that chosen in previous work on CoV_2_O_4_^35^ and MnV_2_O_4_^36,37^. The spin-orbit interaction was included. The projector augmented wave (PAW) potentials^38,39^ explicitly include 13 valenced electrons for Mn ($3{p}^{6}3{d}^{5}4{s}^{2}$), 9 for Co ($3{d}^{8}4{s}^{1}$), 13 for V ($3{s}^{2}3{p}^{6}3{d}^{4}4{s}^{1}$), and 6 for oxygen ($2{s}^{2}2{p}^{4}$). The wave functions were expanded in a plane-wave basis with an energy cutoff of 500 eV. To evaluate the on-site single-ion anisotropy (SIA) interaction *D*, only one cation of interest was kept while the surrounding magnetic atoms were replaced by neutral and isoelectronic Ca^2+^ and Al^3+^ cations for Co^2+^/Mn^2+^ and V^3+^, respectively. This is the same technique that was successfully used for BiFeO_3_^40^ and CaMn_7_O_12_^41^.

### Microscopic spin model

Spin states in spinels can be described by the following model Hamiltonian,

1 $$H=-\frac{1}{2}\sum _{i,j}{J}_{ij}{{\boldsymbol{S}}}_{i}\cdot {{\boldsymbol{S}}}_{j}+\sum _{i}{D}_{i}{({\hat{u}}_{i}\cdot {{\boldsymbol{S}}}_{i})}^{2}$$

which contains six inequivalent sublattices. Isotropic exchange constants ${J}_{{\rm{Co}}-{\rm{V}}}$ describe nearest-neighbor interactions between the Co and V sites. *J*_Co−Co_ and *J*_V−V_ describe nearest-neighbor interactions between Co-sites and V-sites, respectively. The easy-axis anisotropy is assumed to be zero for the Co-sites, while for the B-site spins, the easy-axis anisotropy *D*_v_ is along the local <111> direction. The azimuthal directions of each vanadium spin is constrained, but the canting angle *θ*_*i*_, described in Fig. 4, is allowed to vary between 0 and 2*π*. Since *θ*_*i*_ may have a unique value in adjacent planes, both the two-in-two-out and all-in-all-out configurations are possible. These angles are equal to the polar angle when *θ*_*i*_ is between 0 and *π*, while the polar angles equals $2\pi -{\theta }_{i}$ and the azimuthal angle changes by *π* when *θ*_*i*_ is greater than *π*.

The ground state spin configuration was found by minimizing the classical energy for a given set of parameters. To avoid local minima, this was accomplished by calculating the classical energy on a grid with ${\theta }_{i}=0$ to 2*π* and finding the two angles with the lowest energy. This process was repeated for values of the external magnetic field ranging from 0 to 173 T.

The inelastic neutron cross section for undamped spin waves was calculated using the 1/*S* formalism outlined in ref.^42^ and the appendices of ref.^43^. For direct comparison with experimental intensities, the effects of the magnetic form factor and the instrumental resolution were included in the calculation. The coefficients for Co^2+^, and V^3+^ are from ref.^44^. The resolution function was approximated as a Gaussian in energy with a full width at half-maximum of 1.5 meV. Effects from finite resolution in *Q* were not considered.

While DFT can provide guidance for the values of the isotropic exchange interactions, LSDA + *U* overestimates the experimental moment (M_v_ = 0.47(4)*μ*_*B*_) of CoV_2_O_4_ measured by neutron scattering. Our spin model uses the magnetic moment (M_v_ = 0.5 *μ*_*B*_), which is within the experimental uncertainty. In addition, parameters calculated with DFT were adjusted to reproduce the measured canting angle of CoV_2_O_4_ (*θ* = 20.8 ± 1.7°) in zero field. Care was also taken to avoid a long-range spiral configuration^45^ that was not observed in our neutron diffraction measurements. The final set of parameters used for CoV_2_O_4_ are ${J}_{{\rm{Co}}-{\rm{Co}}}=0.5\,{\rm{meV}}$, ${J}_{{\rm{Co}}-{\rm{V}}}=-2.5\,{\rm{meV}}$, ${J}_{{\rm{V}}-{\rm{V}}}=-11.0\,{\rm{meV}}$, ${D}_{{\rm{V}}}=-2.7\,{\rm{meV}}$, $red{M}_{{\rm{Co}}}=$ 3.00 *μ*_*B*_ and $red{M}_{{\rm{V}}}=0.5\,{\mu }_{B}$.

### Canting angles of the new phase with *D*_v_ = 0.0

At high pressures, we take *D*_v_ = 0.0 in Eq.(1). Then, the total energy per unit magnetic unit cell is

2 $$E(\alpha )=-12{J}_{{\rm{C}}o-V}{S}_{{\rm{C}}o}{S}_{{\rm{V}}}\alpha -4{J}_{{\rm{V}}-V}{S}_{{\rm{V}}}^{2}{\alpha }^{2}+{\rm{constant}}$$

where $\alpha =\,\cos \,{\theta }_{1}+\,\cos \,{\theta }_{2}$. The total energy is at a minimum if

3 $$\alpha =-\frac{3{J}_{{\rm{C}}o-V}{S}_{{\rm{C}}o}}{2{J}_{{V}-V}{S}_{{V}}}$$

which limits the allowed combination of *θ*_1_ and *θ*_2_. When *θ*_1_ = *θ*_2_ and *D*_*Co*_ = *D*_v_ = 0.0, this condition is identical to the expression for *θ* in ref.^36^.

## Electronic supplementary material

Supplementary Information 
