# Supplementary material for: Magnetic Frustration Driven by Itinerancy in Spinel CoV2O4
Source: Sci Rep. 2017 Dec 7;7:17129. doi: 10.1038/s41598-017-17160-0 (PMC5719412; doi:10.1038/s41598-017-17160-0)
Supplement: Supplementary file 1 — Supplementary Information [file 41598_2017_17160_MOESM1_ESM.pdf]

Magnetic Frustration Driven by Itinerancy in Spinel CoV_2O_4

J. H. Lee,[1,*] J. Ma[2,3], S. E. Hahn,[4,5], H. B. Cao,[5] M. Lee,[1] Tao Hong,[6] H.-J. Lee[1], M. S. Yeom,[6,†] S. Okamoto,[7] H. D. Zhou,[8] M. Matsuda,[5] and R. S. Fishman[7]

1. School of Energy and Chemical Engineering, Ulsan National Institute of Science and Technology, Ulsan 44919, Republic of Korea
2. Department of Physics and Astronomy, Shanghai Jiao Tong University, Shanghai 200240, China
3. Key Laboratory of Artificial Structures and Quantum Control, School of Physics and Astronomy, Shanghai Jiao Tong University, Shanghai 200240, China
4. Neutron Data Analysis and Visualization Division, Oak Ridge National Laboratory, Oak Ridge, Tennessee 37831, USA
5. Quantum Condensed Matter Division, Oak Ridge National Laboratory, Oak Ridge, Tennessee 37831, USA
6. Department of Applied Research and Network R&D, Center for Computational Science and Engineering, Division of National Supercomputing R&D, Korea Institute of Science and Technology Information (KISTI), 245 Daehak-ro, Daejeon 34141, Republic of Korea
7. Materials Science and Technology Division, Oak Ridge National Laboratory, Oak Ridge, Tennessee, 37831, USA
8. Department of Physics and Astronomy, University of Tennessee, Knoxville, Tennessee 37996, USA

[*]junhee@unist.ac.kr
[†]msyeom@kisti.re.kr

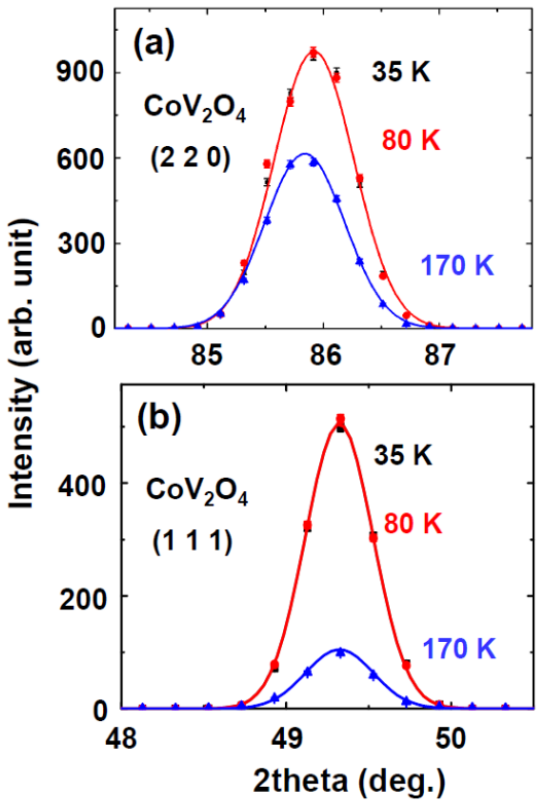

Fig.1 Temperature dependence of neutron theta-to-theta at (220) (a) and (111) (b) Bragg peaks in CoV_2O_4.

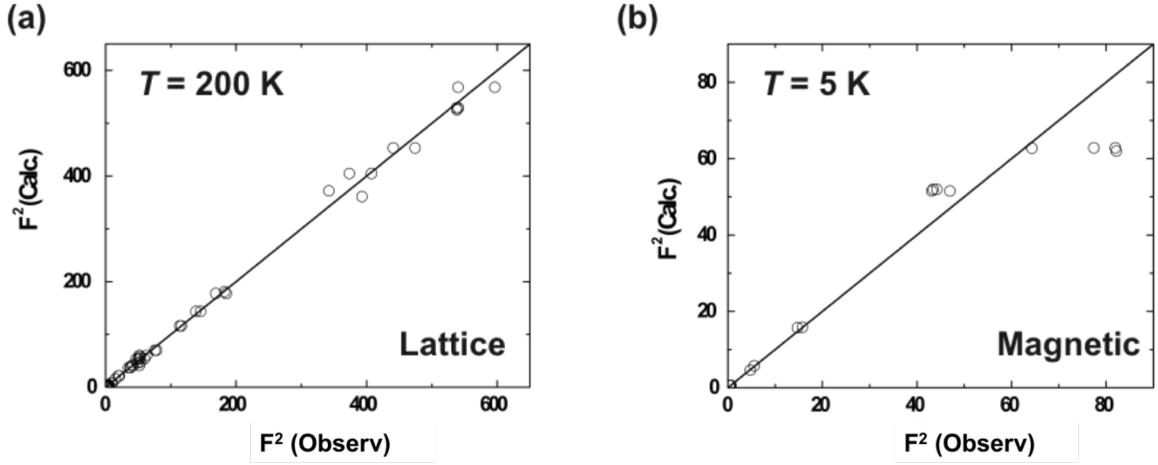

Fig.2 Calculated versus observed squared structure factors (F^2) are plotted to show the fitting quality of (a) nuclear structure refinement at 200 K and (b) magnetic structure refinement *T* = 5 K.
